# Water Quality Prediction Method Based on Multi-Source Transfer Learning for Water Environmental IoT System

**DOI:** 10.3390/s21217271

**Published:** 2021-11-01

**Authors:** Jian Zhou, Jian Wang, Yang Chen, Xin Li, Yong Xie

**Affiliations:** 1 College of Computer, Nanjing University of Posts and Telecommunications, Nanjing 210003, China; b18030412@njupt.edu.cn (J.W.); chenyang_njupt@126.com (Y.C.); xinli@njupt.edu.cn (X.L.); yongxie@njupt.edu.cn (Y.X.); 2 Jiangsu High Technology Research Key Laboratory for Wireless Sensor Networks, Nanjing 210003, China

**Keywords:** water quality prediction, multi-source transfer learning, echo state network, adjacency effect, distributed computing, environmental IoT system

## Abstract

Water environmental Internet of Things (IoT) system, which is composed of multiple monitoring points equipped with various water quality IoT devices, provides the possibility for accurate water quality prediction. In the same water area, water flows and exchanges between multiple monitoring points, resulting in an adjacency effect in the water quality information. However, traditional water quality prediction methods only use the water quality information of one monitoring point, ignoring the information of nearby monitoring points. In this paper, we propose a water quality prediction method based on multi-source transfer learning for a water environmental IoT system, in order to effectively use the water quality information of nearby monitoring points to improve the prediction accuracy. First, a water quality prediction framework based on multi-source transfer learning is constructed. Specifically, the common features in water quality samples of multiple nearby monitoring points and target monitoring points are extracted and then aligned. According to the aligned features of water quality samples, the water quality prediction models based on an echo state network at multiple nearby monitoring points are established with distributed computing, and then the prediction results of distributed water quality prediction models are integrated. Second, the prediction parameters of multi-source transfer learning are optimized. Specifically, the back propagates population deviation based on multiple iterations, reducing the feature alignment bias and the model alignment bias to improve the prediction accuracy. Finally, the proposed method is applied in the actual water quality dataset of Hong Kong. The experimental results demonstrate that the proposed method can make full use of the water quality information of multiple nearby monitoring points to train several water quality prediction models and reduce the prediction bias.

## 1. Introduction

As an important part of the natural environment, water environment plays a vital role in human life. With the rapid development of industry, the discharge of industrial wastewater has increased day by day [1], leading to the deterioration of water environment, and water environment protection is facing severe challenges. Accurate water quality prediction is the basis for water environment protection. The monitoring points are equipped with various water quality Internet of Things (IoT) devices to build the water environmental IoT system [2], which can collect water quality information in real time, making the prediction of accurate water quality possible.

Traditional methods of water quality prediction can be classified into three types: regression analysis, grey systems, and neural networks [3]. Water quality prediction method based on regression analysis is derived from mathematical statistics. It determines the relationship between the dependent variable and the independent variable through the analysis of statistical data, and calculates the correlation coefficient through a certain algorithm, thereby constructing a regression equation to predict water quality information. Ratko et al. [4] proposed a water temperature prediction method based on Gaussian process regression to predict the daily average water temperature of the river. Anja et al. [5] proposed a water quality prediction method based on partial least square regression analysis to predict the water quality information of mining wastewater. Mohammad et al. [6] proposed a prediction method based on M5 model tree and multiple adaptive regression to predict the daily river flow. Water quality prediction method based on grey systems regards the water environment system as a grey system. After that, a strong regular series for water quality prediction is generated by identifying the relationships of system factors. Zhang et al. [7] constructed a grey prediction model to predict the chemical oxygen of industrial wastewater. Yang et al. [8] constructed a GM (1,1) model to predict the water quality information of the lake. Xue et al. [9] constructed a grey prediction model to predict the mineralization of groundwater. Xiao et al. [10] applied grey theory to construct a model to predict the affecting factors of water bloom. Water quality prediction method based on neural networks forms an adaptive nonlinear system through the connection of neurons, using the neural networks to adaptively learn the trend of water quality information. With the emergence of cloud computing, edge computing and other technologies [11,12,13], neural networks requiring complex computation were gradually applied to water quality prediction. Dawood et al. [14] constructed an artificial neural network to predict the water quality information. Zhou et al. [15] proposed a water quality prediction method based on improved grey relational analysis and long-short term memory (LSTM) neural network to predict the dissolved oxygen. Dong et al. [16] proposed a water quality prediction method based on Savitzky-Golay and LSTM to predict the water quality information. Hu et al. [17] constructed a deep LSTM to predict pH and water temperature. Considering the temporality and the nonlinearity of water quality information, neural networks have more advantages and better prediction performance than the other two types of methods but require a large number of training samples. If the target monitoring point has too few training samples, the accuracy of water quality prediction will be reduced.

Water flows and exchanges between multiple monitoring points [18] in the same water area result adjacency effect in their water quality information. The prediction accuracy of neural networks can be improved if the adjacency effect is used for neural networks. Traditional transfer learning methods, such as transfer component analysis (TCA) [19], are usually used for single-source transfer, which can transfer the features of water quality samples from a single nearby monitoring point to a target monitoring point [20]. However, TCA does not consider the bias between the features of water quality samples of multiple nearby monitoring points, which makes it not applicable for the transfer of water quality samples of multiple nearby monitoring points. Target monitoring points are often surrounded with multiple nearby monitoring points in practice. Compared with traditional transfer learning methods, which can only effectively use one source domain, multi-source transfer learning (MSTL) [21,22] can make full use of multiple source domains. Therefore, we proposed a water quality prediction framework based on MSTL, effectively using the water quality information of multiple nearby monitoring points with distributed computing.

The water quality information changes periodically along with time, so it has the nature of temporality. By using the temporality, the accuracy of water quality prediction can be effectively improved. Echo state network (ESN) [23], as an improved model of recurrent neural network (RNN) [24], retains the information left at the last moment through the internal connections of reservoir, which can effectively use the temporality. Moreover, ESN only needs to use the linear regression algorithm to train the output weights, which can solve the problem of slow convergence speed of traditional RNN. Therefore, we establish the distributed water quality prediction models based on ESN at multiple nearby monitoring points in the framework, effectively using the temporality of water quality information.

Bias [25] exists not only in the feature alignment of nearby monitoring points and target monitoring points, but also in the model alignment of the water quality prediction models at multiple nearby monitoring points. Therefore, we optimize the prediction parameters of MSTL to improve the prediction accuracy of the models.

In this paper, we propose a water quality prediction method based on MSTL, for the purpose of making full use of the adjacency effect of water quality information. The contributions of this paper are listed as follows.

(1) We construct a water quality prediction framework based on MSTL. In particular, the common features of water quality samples of multiple nearby monitoring points and the target monitoring point are extracted and then aligned. Afterwards, according to the aligned features of water quality samples, the water quality prediction models based on ESN at multiple nearby monitoring points are established with distributed computing, and then the prediction results of distributed water quality prediction models are integrated. This framework successfully solves the problem of an insufficient number of training samples of the target monitoring point.
(2) We optimize the prediction parameters of MSTL. In particular, the back propagates the population deviation based on multiple iterations and can reduce the feature alignment bias and the model alignment bias to improve the prediction accuracy of the models.
(3) We perform experiments in the actual water quality dataset of Hong Kong. The experimental results demonstrate that the proposed method can train multiple water quality prediction models by using the adjacency effect, and thus reduce the prediction bias and improve the prediction accuracy compared with other similar methods.

The rest of this paper is organized as follows. Section 2 gives the details of the proposed method, including the water quality prediction framework based on MSTL, the prediction parameters optimization of MSTL, and the overall process. Section 3 gives the experimental results and analyses. Section 4 is the summary of this paper.

## 2. Methods

### 2.1. Water Quality Prediction Framework Based on MSTL

**Figure 1:**
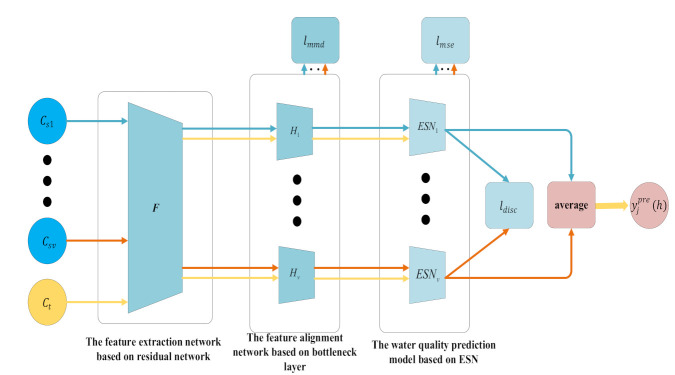
Water quality prediction framework based on MSTL.

We construct a water quality prediction framework based on MSTL, as shown in Figure 1. First, we use the feature extraction network based on the residual network [26] to extract the water quality features of nearby monitoring points and the target monitoring point into the same feature space, to obtain the common features of water quality samples of nearby monitoring points and the target monitoring point. Second, we use the feature alignment networks based on a bottleneck layer [27] to align the common features of water quality samples in the same feature space, to obtain the aligned features. Third, we establish the water quality prediction model based on ESN at every nearby monitoring point with distributed computing and predict the water quality information at the next moment according to the aligned features of water quality samples. Finally, we integrate the results of distributed water quality prediction models to reduce the prediction bias.

If there are v nearby monitoring points around the target monitoring point, respectively construct the features of water quality samples of the j-th nearby monitoring point and the target monitoring point at the previous n moments as

(1) $$C_{sj}=\left\{c_{sj}\left(d+1\right),c_{sj}\left(d+2\right),\cdots ,c_{sj}\left(h\right),\cdots ,c_{sj}\left(n\right)\right\}$$

(2) $$C_{t}=\left\{c_{t}\left(d+1\right),c_{t}\left(d+2\right),\cdots ,c_{t}\left(h\right),\cdots ,c_{t}\left(n\right)\right\}$$

where $c_{sj}\left(h\right)=\left\{x_{sj}\left(h-1\right),x_{sj}\left(h-2\right),\cdots ,x_{sj}\left(h-d\right)\right\}$ and $c_{t}\left(h\right)=\left\{x_{t}\left(h-1\right),x_{t}\left(h-2\right),\cdots ,x_{t}\left(h-d\right)\right\}$ represent the features of water quality samples of the j-th nearby monitoring point and the target monitoring point at the h-th moment, respectively. In particular, d represents the size of the sliding window, $x_{sj}\left(h-1\right)$ and $x_{t}\left(h-1\right)$ represent the water quality information of the j-th nearby monitoring point and the target monitoring point at the (h−1)-th moment, respectively.

**Figure 2:**
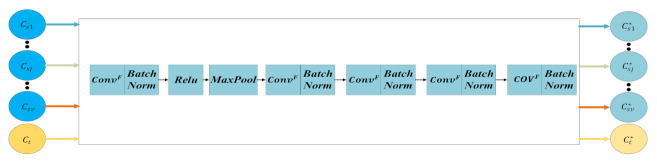
Structure of feature extraction network based on residual network.

First, we construct the feature extraction network based on residual network (F), for the purpose of extracting the common features of water quality samples of v nearby monitoring points and the target monitoring point. The structure of this network is shown in Figure 2.

In Figure 2, $Conv^{F}$ is the convolution kernel, BatchNorm is the normalization algorithm, Rule is the activation function, and MaxPool is the max pooling layer. The features of water quality samples extracted from the j-th nearby monitoring point and the target monitoring point are respectively $C_{sj}^{*}$ and $C_{t}^{*}$, and they are calculated by

(3) $$C_{sj}^{*}=F\left(C_{sj}\right)$$

(4) $$C_{t}^{*}=F\left(C_{t}\right)$$

**Figure 3:**
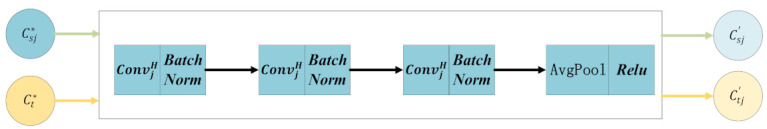
Structure of the feature alignment network based on the bottleneck layer at the j-th nearby monitoring point.

Second, we construct the feature alignment networks based on the bottleneck layer ($\left\{H_{1},H_{2},\cdots ,H_{j},\cdots ,H_{v}\right\}$) at v nearby monitoring points, for the purpose of aligning the common features extracted from the nearby monitoring point with the features extracted from the target monitoring point. In particular, $H_{j}$ is the feature alignment network at the j-th nearby monitoring point, and its structure is shown in Figure 3.

In Figure 3, $Conv_{j}^{H}$ is the convolution kernel, Rule is the activation function, and AvgPool is the average pooling layer. The aligned features of water quality sample of the j-th nearby monitoring point and the target monitoring point are respectively $C_{sj}^{’}$ and $C_{tj}^{’}$, and they are calculated by

(5) $$C_{sj}^{’}=H_{j}\left(C_{sj}^{*}\right)$$

(6) $$C_{tj}^{’}=H_{j}\left(C_{t}^{*}\right)$$

After aligning the common features of water quality samples, construct the water quality sample sets of the j-th nearby monitoring point and target monitoring point as $U_{sj}=\left\{u_{sj}\left(d+1\right),u_{sj}\left(d+2\right),\cdots ,u_{sj}\left(h\right),\cdots ,u_{sj}\left(n\right)\right\}$ and $U_{tj}=\left\{u_{tj}\left(d+1\right),u_{tj}\left(d+2\right),\right.\cdots ,u_{tj}\left(h\right),\left.\cdots ,u_{tj}\left(m\right)\right\}$, respectively. In particular, $u_{sj}\left(h\right)=\left\{c_{sj}^{’}\left(h\right),y_{sj}\left(h\right)\right\}$ and $u_{tj}\left(h\right)=\left\{c_{tj}^{’}\left(h\right),y_{t}\left(h\right)\right\}$ respectively represent the water quality samples of the j-th nearby monitoring point and the target monitoring point at the h-th moment, where $c_{sj}^{’}\left(h\right)$ and $c_{tj}^{’}\left(h\right)$ are the aligned feature of water quality sample of the j-th nearby monitoring point and the target monitoring point at the h-th moment. $y_{sj}\left(h\right)$ and $y_{t}\left(h\right)$ are the real water quality information of the j-th nearby monitoring point and the target monitoring point at the h-th moment.

We combine $U_{sj}$ and $U_{tj}$ to obtain the water quality sample set $U_{j}^{train}=U_{sj}\cup U_{tj}=\left\{u_{j}^{train}\left(d+1\right),\right.\left.u_{j}^{train}\left(d+2\right),\cdots ,u_{j}^{train}\left(h\right),\cdots ,u_{j}^{train}\left(m+n-d\right)\right\}$, where $u_{j}^{train}\left(h\right)=\left\{c_{j}^{total}\left(h\right),\right.\left.y_{j}^{total}\left(h\right)\right\}$ is the water quality sample at the h-th moment, $c_{j}^{total}\left(h\right)$ is the feature of water quality sample at the h-th moment, and $y_{j}^{total}\left(h\right)$ is the real water quality information at the h-th moment. $U_{j}^{train}$ will be used to train the following water quality prediction model.

**Figure 4:**
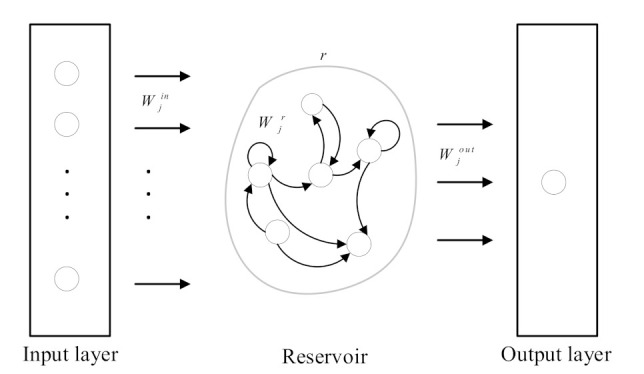
Structure of water quality prediction model based on ESN.

Afterwards, we construct the water quality prediction models based on ESN at v nearby monitoring points ($\left\{ESN_{1},ESN_{2},\cdots ,ESN_{j},\cdots ,ESN_{v}\right\}$), where $ESN_{j}$ is the distributed water quality prediction model at the j-th nearby monitoring point, and its structure is shown in Figure 4. The model consists of an input layer with d neurons, a reservoir with r neurons, and an output layer with one neuron. Besides, the input of the model is the feature of water quality sample at the h-th moment ($c_{j}^{total}\left(h\right)$), and the output is the predicted water quality information at the h-th moment ($y_{j}^{pre}\left(h\right)$).

The calculation of the water quality prediction model based on ESN at the j-th nearby monitoring point is as

(7) $$s_{j}\left(h\right)=Tanh\left(W_{j}^{r}s_{j}\left(h-d\right)+W_{j}^{in}c_{j}^{total}\left(h\right)\right)$$

(8) $$y_{j}^{pre}\left(h\right)=W_{j}^{out}s_{j}\left(h\right)$$

where Tanh is the activation function, and $s_{j}\left(h\right)$ is the internal state vector of the reservoir. $W_{j}^{in}$ is the input layer weight, $W_{j}^{r}$ is the reservoir weight, and $W_{j}^{out}$ is the output weight. In particular, $W_{j}^{out}$ is trained by the ridge regression algorithm [28] according to $U_{j}^{train}$, $W_{j}^{r}$ and is scaled by

(9) $$W_{j}^{r}=\alpha \frac{1}{\left|\rho \right|}W_{0}$$

where α is the scaling range and 0<α<1. ρ is the spectral radius of $W_{j}^{r}$, and $W_{0}$ is a sparse matrix which is randomly generated.

Finally, we integrate the prediction results of distributed water quality prediction models at multiple nearby monitoring points to obtain the final prediction result ($y^{pre}\left(h\right)$) by using the arithmetic average. $y^{pre}\left(h\right)$ is calculated by

(10) $$y^{pre}\left(h\right)=\frac{1}{v}\sum_{j=1}^{v}y_{j}^{pre}\left(h\right)$$

### 2.2. Prediction Parameters Optimization of MSTL

We optimize the prediction parameters of MSTL to reduce the feature alignment bias between nearby monitoring points and the target monitoring point, and to minimize the model alignment bias between the water quality prediction models. Specifically, to minimize the overall bias ($l_{total}$), $CONV^{F}$, $CONV_{j}^{H}$ and $W_{j}^{out}$ are updated by the stochastic gradient descent (SGD), since $W_{j}^{out}$ affects the prediction results of the water quality prediction model, $CONV^{F}$ and $CONV_{j}^{H}$ affect the aligned features obtained by the feature alignment networks. The smaller $l_{total}$ is, the better the prediction accuracy is. $l_{total}$ is calculated by

(11) $$l_{total}=l_{mse}+\lambda \left(l_{mmd}+l_{disc}\right)$$

where λ is the trade-off parameter, which is used to measure the importance of $l_{mmd}$ and $l_{disc}$. λ is calculated by

(12) $$\lambda =\frac{2}{1+e^{\frac{10i}{iter}}}-1$$

where iter is the total number of iterations, and i is the current number of iterations.

$l_{mse}$ is the model prediction bias of the water quality prediction models at the v nearby monitoring points. The smaller $l_{mse}$ is, the smaller the model prediction bias is. $l_{mse}$ is calculated by

(13) $$l_{mse}=\frac{1}{v}\sum_{j=1}^{v}Mse\left(y_{j}^{pre}\left(h\right),y^{true}\left(h\right)\right)$$

where $y_{j}^{pre}\left(h\right)$ is the predicted water quality information of the prediction model at the j-th nearby monitoring point, $y^{true}$ is the real water quality information, and Mse is the mean square error function.

$l_{mmd}$ is the feature alignment bias between nearby monitoring points and the target monitoring point. The smaller $l_{mmd}$ is, the smaller the feature alignment bias is. $l_{mmd}$ is calculated by

(14) $$l_{mmd}=\frac{1}{v}\sum_{j=1}^{v}MMD\left(C_{sj}^{’}\left(h\right),C_{tj}^{’}\left(h\right)\right)$$

where MMD is the maximum mean discrepancy function [29], which is used to measure the distance between the aligned features of water quality samples of nearby monitoring points and the target monitoring point after mapping to the same feature space.

$l_{disc}$ is the model alignment bias between the water quality prediction models at multiple nearby monitoring points. The smaller the $l_{disc}$ is, the smaller the model alignment bias is. $l_{disc}$ is calculated by

(15) $$l_{disc}=\frac{2}{\left(v-1\right)v}\sum_{j=1}^{v-1}\sum_{i=j+1}^{v}\left(\left|y_{i}^{pre}\left(h\right)-y_{j}^{pre}\left(h\right)\right|\right)$$

### 2.3. Process of Water Quality Prediction Method Based on MSTL

**Figure 5:**
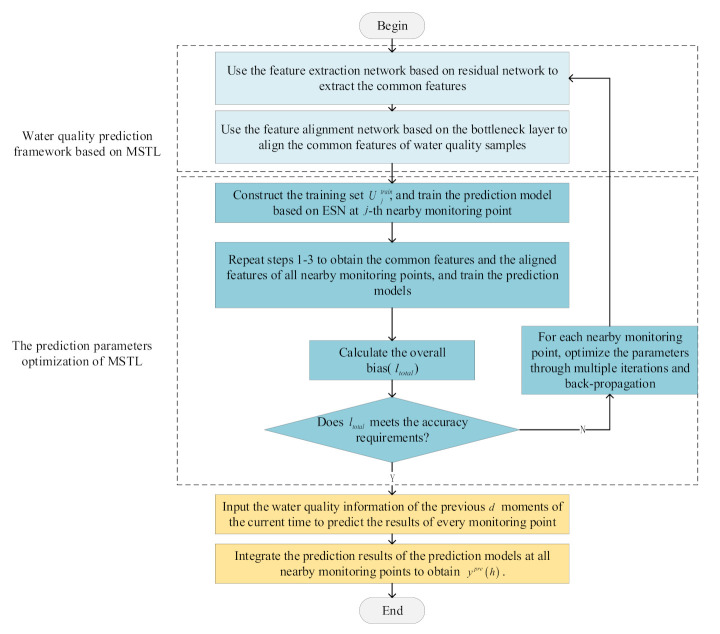
Overall process of water quality prediction method based on MSTL.

The overall process of the water quality prediction method based on MSTL is summarized in Figure 5. The specific steps are as follows:

Step 1: Use the feature extraction network based on residual network to extract the common features of water quality samples of the j-th nearby monitoring point and target monitoring point ($C_{sj}^{*}$ and $C_{t}^{*}$).

Step 2: Use the feature alignment network based on the bottleneck layer of the j-th nearby monitoring point to align the common features of water quality samples of the j-th nearby monitoring point and the target monitoring point ($C_{sj}^{’}$ and $C_{tj}^{’}$).

Step 3: Construct the training set $U_{j}^{train}$, and train the distributed water quality prediction model based on ESN at the j-th nearby monitoring point.

Step 4: Repeat Steps 1–3 to obtain the common features and the aligned features of water quality samples of all nearby monitoring points, and train distributed water quality prediction models based on ESN at all nearby monitoring points.

Step 5: Calculate the overall bias ($l_{total}$) according to the aligned features of water quality samples of all nearby monitoring points and the prediction results of distributed water quality prediction models based on ESN.

Step 6: Judge whether $l_{total}$ meets the accuracy requirements. If the requirements are met, go to step 8. Otherwise, go to step 7.

Step 7: For every nearby monitoring point, update $CONV^{F}$, $CONV_{j}^{H}$, and $W_{j}^{out}$ through multiple iterations and back-propagating $l_{total}$,. After that, go to step 1.

Step 8: At the j-th nearby monitoring point, input the water quality information of the previous d moments of the current time of the target monitoring point into the optimized water quality prediction framework based on MSTL, then obtain the prediction result through distributed computing. In the same way, the prediction results of distributed water quality prediction models of all nearby monitoring points are obtained.

Step 9: Integrate the prediction results of distributed water quality prediction models at all nearby monitoring points to obtain the final prediction result ($y^{pre}\left(h\right)$).

## 3. Experimental Results and Analyses

The proposed method is implemented by Python and Torch. First, we describe the specific dataset of the experiments. Second, we select the prediction parameters of MSTL. Afterwards, MSTL is compared with other transfer methods. Finally, we compare ESN with other prediction models.

We set 20% samples of the target monitoring point as the test sample set and 20% as the validation sample set. Thus, the training sample set is composed of the remaining 60% samples of the target monitoring point and the samples transferred from nearby monitoring points. The mean squared error (MSE) is chosen as the indicator measuring the prediction bias. The smaller MSE is, the smaller the prediction bias is. Specifically, MSE is calculated by

(16) $$MSE=\frac{1}{q}\sum_{t=1}^{q}\left(y^{true}\left(t\right)-y^{pre}\left(t\right)\right)$$

where q is the number of samples, $y^{true}$ is the real water quality information, and $y^{pre}$ is the predicted water quality information.

### 3.1. Datasets

**Figure 6:**
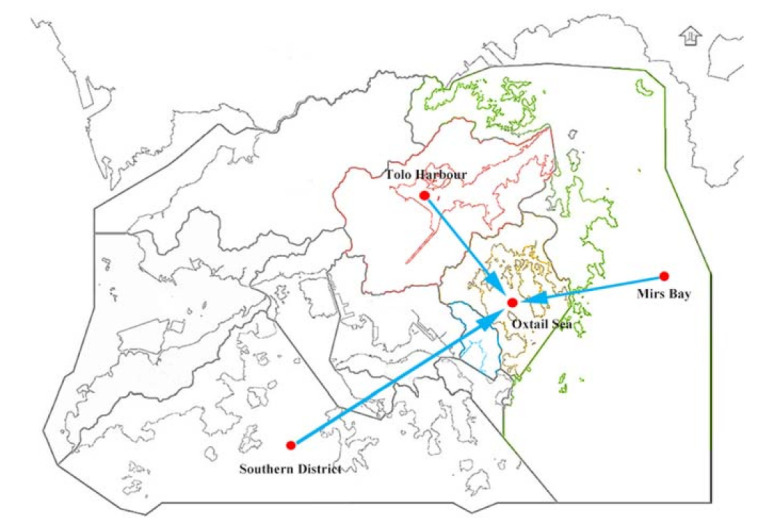
Spatial location of Oxtail Sea and nearby monitoring points.

We performed two experiments. In the first experiment, we set Oxtail Sea as the target monitoring point. Oxtail Sea has only 3193 pieces of water quality information, which is slightly insufficient. The spatial location of monitoring points in the first experiment is shown in Figure 6. Tolo Harbour, Mirs Bay and Southern District are close to Oxtail Sea in water area, and they have the adjacency effect. As a result, we consider these three locations as nearby monitoring points. Afterwards, we use the framework based on MSTL to align the features of water quality samples of these three nearby monitoring points with Oxtail Sea, and then use the samples of these three nearby monitoring points to train three water quality prediction models. Among them, Tolo Harbour has 3192 pieces of water quality information, Mirs Bay has only 758 pieces of water quality information, and Southern District has 4467 pieces of water quality information. The water quality indicators of Oxtail Sea include dissolved oxygen (DO), phosphate, water temperature (WT), and nitrite. The data of these indicators are collected by the sensors and transmitted back approximately every fifteen days. The purpose of the experiment is to predict the DO of Oxtail Sea at the next moment.

**Figure 7:**
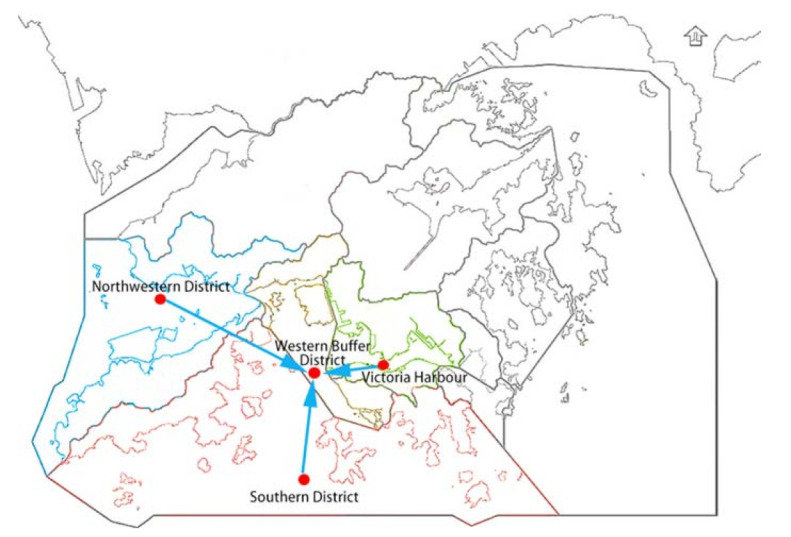
Spatial location of Western Buffer District and nearby monitoring points.

In the second experiment, we set the Western Buffer District as the target monitoring point. Western Buffer District has only 1440 pieces of water quality information, which is also slightly insufficient. The spatial location of monitoring points in the second experiment is shown in Figure 7. Figure 7 shows the Northwestern District, the Southern District and the Victoria Harbour are close to Western Buffer District in water area and they have the adjacency effect. As a result, we consider these three locations as nearby monitoring points. The experimental procedure is the same as the first experiment. Among them, the Northwestern District has only 1630 pieces of water quality information, the Southern District has 4467 pieces of water quality information, and the Victoria Harbour has 4158 pieces of water quality information. The water quality indicators and the prediction purpose of the Western Buffer District are the same as the first experiment.

### 3.2. Parameters Selection

In order to improve the prediction accuracy, we select the parameters including the size of sliding window (d), the size of reservoir (r) and the size of spectral radius (ρ) in the water quality prediction models based on ESN. In the experiment of Oxtail Sea, $l_{total}$ converges when the number of iterations is 300. Table 1 shows the prediction results of distributed water quality prediction models based on ESN with different parameters in Oxtail Sea. When d=3, r=500, and ρ=0.7, the prediction result of Oxtail Sea is the best ($l_{total}=0.085$, MSE=0.0060). Similarly, in the experiment of the Western Buffer District, $l_{total}$ converges also when the number of iterations is 300. Table 2 shows the prediction results of distributed water quality prediction models based on ESN with different parameters in the Western Buffer District. When d=3, r=500, and ρ=0.6, the prediction result of the Western Buffer District is the best ($l_{total}=0.0111$, MSE=0.0106). The optimal parameters mentioned above are used in the subsequent experiments.

### 3.3. Comparison of Transfer Methods

We compare MSTL with non-expansion, TCA [20] and the joint class proportion and optimal transport (JCPOT) [30]. In particular, non-expansion uses only the water quality information of the target monitoring point. TCA transfers the features of water quality samples from a single nearby monitoring point to the target monitoring point, and selects the high-quality samples based on similarity and time sequence. JCPOT predicts the water quality information by using the optimal transport to correct and align the feature alignment bias between multiple source domains and target domain. MSTL extracts and aligns the features of water quality samples of multiple nearby monitoring points and the target monitoring point and trains the model through the aligned samples. The prediction results of different transfer methods in the Oxtail Sea and the Western Buffer District locations are shown in Table 3.

From Table 3, we can observe that the prediction bias of MSTL is lower than that of non-expansion either in the Oxtail Sea or in the Western Buffer District. Besides, the prediction bias of MSTL is lower than that of TCA and JCPOT, because TCA can only use the water quality information of a single nearby monitoring point and JCPOT does not consider the effect of the feature alignment bias between different source domains. The prediction results show that MSTL can effectively use the water quality information of nearby monitoring points to train multiple water quality prediction models, which can reduce the model prediction bias and improve the prediction accuracy.

### 3.4. Comparison of Prediction Models

**Figure 8:**
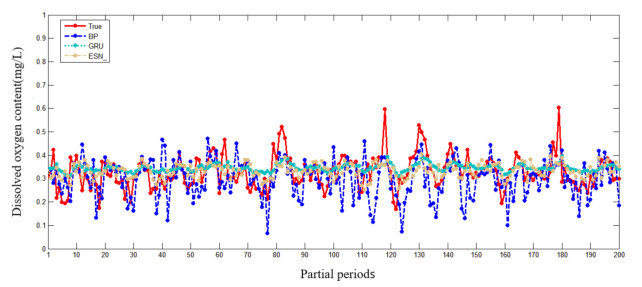
Partial prediction results of different prediction models in Oxtail Sea.

**Figure 9:**
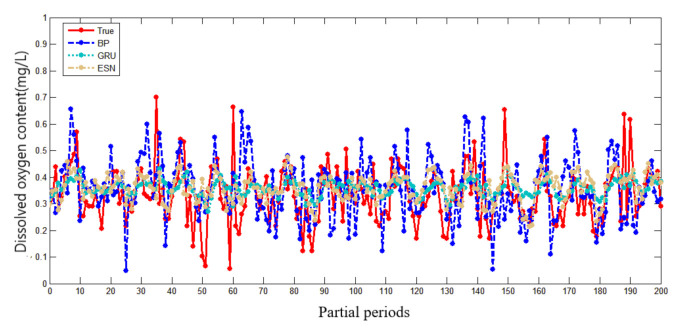
Partial prediction results of different prediction models in Western Buffer District.

**Figure 10:**
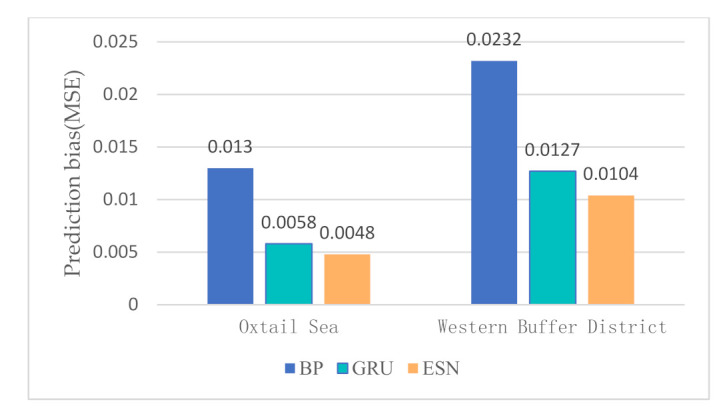
Comparison of prediction bias of different water quality prediction models.

**Figure 11:**
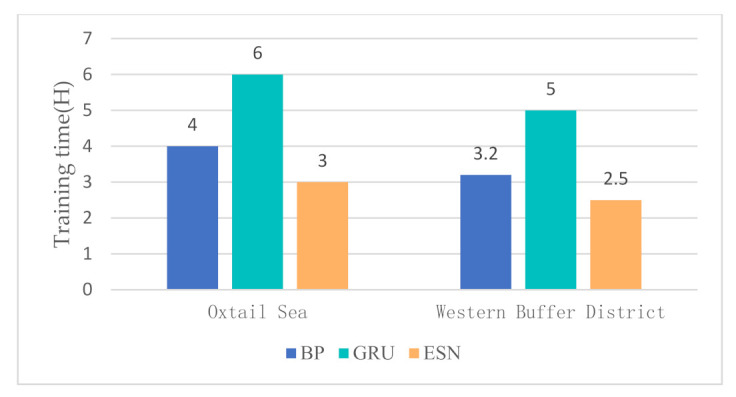
Comparison of training time of different water quality prediction models.

In the water quality prediction framework based on MSTL, we compare the water quality prediction models based on ESN with the water quality prediction models based on back propagation (BP) network, and the water quality prediction models based on gated recurrent unit (GRU) network. Like ESN, both BP and GRU have only one hidden layer. As a widely used basic neural network, BP has the advantages of simple structure and small calculation. As an improvement of LSTM, GRU adds a gating mechanism to make it have a memory ability. Compared with BP, the training of GRU is more complex. Partial prediction results of different prediction models in the Oxtail Sea and the Western Buffer District are shown in Figure 8 and Figure 9, respectively. The comparisons of different water quality prediction models in terms of prediction bias and training time are shown in Figure 10 and Figure 11.

Figure 8 and Figure 9 show that the accuracy of BP is poor, and the prediction results fluctuate greatly. The prediction results of GRU and ESN are close when the data fluctuate slightly. Overall, ESN has better prediction ability than that of GRU either in the peak or in the valley part of the data. Figure 10 and Figure 11 show that ESN has the smallest prediction bias and the shortest training time in the Oxtail Sea or the Western Buffer District, because ESN has a special reservoir structure and use only a simple linear regression algorithm for training.

**Figure 12:**
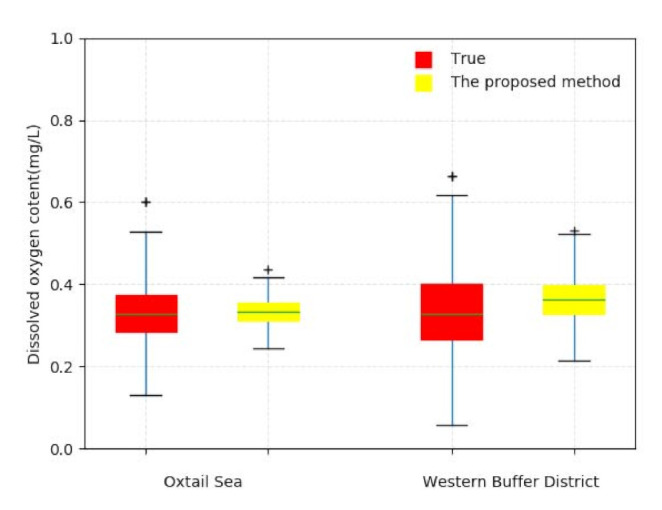
Box-plot comparison of predicted water quality information and real water quality information.

To further illustrate the prediction accuracy of the proposed method, Figure 12 gives the box-plot comparison of the predicted water quality information and the real water quality information in the Oxtail Sea and the Western Buffer District. As seen in the figure, there exists a nearly uniform presentation through the observations of the measures, including the upper and lower quartiles, the upper and lower bound, the median and the outliers.

## 4. Conclusions

Water environmental IoT system, which can collect water quality information in real time, provides the possibility for accurate water quality prediction. In this paper, we propose a water quality prediction method based on MSTL for water environmental IoT system, to effectively use the water quality information of nearby monitoring points, and then improve the prediction accuracy of water quality. First, a water quality prediction framework based on MSTL is constructed, which establishes multiple water quality prediction models based on ESN at multiple nearby monitoring points with distributed computing. Second, the water quality prediction parameters of MSTL are optimized. Specifically, the back propagates population deviation based on multiple iterations reducing the feature alignment bias and the model alignment bias. Finally, the proposed method is compared with other similar methods in the actual water quality dataset of Hong Kong. The experimental results demonstrate that the proposed method can effectively align the features of water quality samples of multiple nearby monitoring points through MSTL and use the aligned samples of multiple nearby monitoring points to train multiple water quality prediction models, which can effectively reduce the prediction bias. It should be noted that the same type of sensors needs to be used at different monitoring points to collect the data of the same water quality indicators, so that the prediction models at different monitoring points have the same input parameter. In the following work, we will study to break through this limitation.

## Figures and Tables

**Table 1:** Prediction results with different parameters in Oxtail Sea. The bold numbers are the best parameters.

d	r	ρ	$l_{total}$	MSE
2	250	0.6	0.092	0.0062
	250	0.7	0.093	0.0064
	250	0.8	0.092	0.0062
	250	0.9	0.091	0.0061
	500	0.6	0.092	0.0063
	500	0.7	0.093	0.0062
	500	0.8	0.092	0.0061
	500	0.9	0.095	0.0065
	750	0.6	0.092	0.0062
	750	0.7	0.093	0.0063
	750	0.8	0.094	0.0064
	750	0.9	0.093	0.0062
**3**	250	0.6	0.089	0.0063
	250	0.7	0.088	0.0062
	250	0.8	0.088	0.0062
	250	0.9	0.087	0.0062
	500	0.6	0.087	0.0061
	**500**	**0.7**	**0.085**	**0.0060**
	500	0.8	0.088	0.0063
	500	0.9	0.086	0.0061
	750	0.6	0.087	0.0062
	750	0.7	0.089	0.0063
	750	0.8	0.089	0.0064
	750	0.9	0.088	0.0063
4	250	0.6	0.093	0.0066
	250	0.7	0.089	0.0062
	250	0.8	0.092	0.0063
	250	0.9	0.090	0.0061
	500	0.6	0.092	0.0063
	500	0.7	0.094	0.0065
	500	0.8	0.093	0.0062
	500	0.9	0.092	0.0061
	750	0.6	0.092	0.0062
	750	0.7	0.093	0.0063
	750	0.8	0.094	0.0064
	750	0.9	0.089	0.0062

**Table 2:** Prediction results with different parameters in the Western Buffer District. The bold numbers are the best parameters.

d	r	ρ	$l_{total}$	MSE
2	250	0.6	0.0113	0.0108
	250	0.7	0.0114	0.0108
	250	0.8	0.0112	0.0107
	250	0.9	0.0115	0.0110
	500	0.6	0.0114	0.0108
	500	0.7	0.0114	0.0109
	500	0.8	0.0115	0.0111
	500	0.9	0.0114	0.0109
	750	0.6	0.0115	0.0110
	750	0.7	0.0116	0.0109
	750	0.8	0.0115	0.0108
	750	0.9	0.0115	0.0109
**3**	250	0.6	0.0116	0.0111
	250	0.7	0.0116	0.0110
	250	0.8	0.0115	0.0109
	250	0.9	0.0115	0.0108
	**500**	**0.6**	**0.0111**	**0.0106**
	500	0.7	0.0113	0.0108
	500	0.8	0.0114	0.0109
	500	0.9	0.0115	0.0109
	750	0.6	0.0115	0.0110
	750	0.7	0.0114	0.0109
	750	0.8	0.0114	0.0108
	750	0.9	0.0115	0.0109
4	250	0.6	0.0113	0.0107
	250	0.7	0.0114	0.0108
	250	0.8	0.0114	0.0108
	250	0.9	0.0115	0.0110
	500	0.6	0.0116	0.0112
	500	0.7	0.0115	0.0111
	500	0.8	0.0114	0.0109
	500	0.9	0.0113	0.0108
	750	0.6	0.0114	0.0110
	750	0.7	0.0115	0.0111
	750	0.8	0.0114	0.0109
	750	0.9	0.0113	0.0108

**Table 3:** Prediction results of different transfer methods in the Oxtail Sea and the Western Buffer District.

Monitoring Points	Method	Nearby Monitoring Point	MSE
Oxtail Sea	non-expansion	non	0.0167
	TCA	Tolo Harbour	0.0118
		Mirs Bay	0.0144
		Southern District	0.0120
	JCPOT	Tolo Harbour, Mirs Bay, Southern District	0.0133
	MSTL	Tolo Harbour, Mirs Bay, Southern District	0.0048
Western Buffer District	non-expansion	non	0.0128
	TCA	Northwestern District	0.0120
		Southern District	0.0125
		Victoria Harbour	0.0118
	JCPOT	Northwestern District, Southern District, Victo- ria Harbour	0.0125
	MSTL	Northwestern District, Southern District, Victo- ria Harbour	0.0104
